# There is No test–retest reliability of brain activation induced by robotic passive hand movement: A functional NIRS study

**DOI:** 10.1002/brb3.1788

**Published:** 2020-08-13

**Authors:** Sungjin Bae, Yonghee Lee, Pyung‐Hun Chang

**Affiliations:** ^1^ Department of Robotics Engineering Graduate School Daegu Gyeongbuk Institute of Science & Technology Daegu Korea; ^2^ Department of Statistics University of Seoul Seoul Korea

**Keywords:** activation map, brain activation, fNIRS, intraclass correlation coefficient, robotic passive movement, test–retest reliability

## Abstract

**Introduction:**

The basic paradigm of rehabilitation is based on the brain plasticity, and for promoting it, test–retest reliability (TRR) of brain activation in which certain area of the brain is repeatedly activated is required. In this study, we investigated whether the robotic passive movement has the TRR of brain activation. While active training has been shown to have TRR, but there still have been arguments over the TRR by passive movement.

**Methods:**

In order to test TRR, 10 repetitive sessions and various intervals (1 day, 3 days, 7 days, 23 days, 15 min, and 6 hr) were applied to five subjects, which had the same statistical power as applying two sessions to 50 subjects. In each session, three robot speeds (0.25, 0.5, and 0.75 Hz) were applied to provide passive movement using the robot. The fNIRS signal (oxy‐Hb) generated in the primary sensorimotor area (SM1) was measured on a total of 29 channels. At this time, we used activation maps and intraclass correlation coefficient (ICC) values to examine the TRR and the effect of robot speeds and intervals on TRR.

**Results:**

As a result, activation maps showed prominent variation regardless of robot speeds and interval, and the ICC value (=0.002) showed no TRR of brain activation for robotic passive movement.

**Conclusion:**

The brain activation induced by the robotic passive movement alone has very poor TRR, suggesting that further enhancement is required to strengthen the TRR by complementing active user engagements.

## Graphical abstract

We investigated the test–retest reliability of activation induced by robotic passive movement. In order to test TRR, 10 repetitive sessions and various intervals (1 day, 3 days, 7 days, 23 days, 15 min, and 6 hr) were applied to five subjects. As a result, our findings show that there is no TRR in robotic passive movement with ICC of 0.002 and session across variability in activation map.

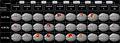

## 1 INTRODUCTION

*Does the brain activation induced by the robotic passive movement have TRR?* Focusing on this simple question, the present paper attempts to provide an answer to it. The importance and background of this question are provided as follows.

The brain plasticity, the ability of the brain system to reorganize its structure and function, is the basic mechanism for restoring motor function in stroke patients (Cramer et al., 2011; Murphy & Corbett, 2009; Schaechter, 2004). Recovery of motor function by brain plasticity is made by modulating the brain activation through the manipulation of the external stimuli (Kaplan, 1988). Such stimuli encompass repeated physical movement training for physical rehabilitation, as was reported in the previous works (Rossini et al., 2007; Takahashi, Der‐Yeghiaian, Le, Motiwala, & Cramer, 2008; Tardy et al., 2006). Specifically, through the steady, active movement training, the change due to the brain plasticity was confirmed by an activation area that had notably expanded (Willer, Ramsay, Wise, Friston, & Frackwiak, 1993). From these findings, we may infer two insights: First, the activation area is likely to serve as an *epicenter* from which the brain plasticity develops; secondly, its *repeated* activation by a movement training appears crucial to promoting brain plasticity. The success in the repeated activation can be evaluated by the test–retest reliability (TRR).

The TRR refers to the consistency of measurement in various science fields (Weir, 2005). Thus, the TRR is evaluated as subjects repeat the experiments over two or more times under the same conditions (Streiner, Norman, & Cairney, 2015). In the neuroimaging field, the TRR of brain activation represents how much likely a certain area is repeatedly activated, as has been manifested by the research works in the following paragraph.

The TRR is different depending on whether the motor task is *active* or *passive*. Regarding *active* movement, many previous studies reported that there is good TRR in brain activation (Bhambhani, Maikala, Farag, & Rowland, 2006; Durduran et al., 2004; Plichta et al., 2007; Sato et al., 2006; Strangman, Goldstein, Rauch, & Stein, 2006). Those works have unanimously supported the existence of strong TRR in active movements. How about the TRR in the passive movement case?

Before we discuss it, it appears necessary to explain why we consider the passive case in the first place, now that the *active* movement has already shown the strong TRR. An important reason is that those patients with severe symptoms who cannot actively move their affected body parts and yet need movement training for recovery have to rely solely on passive movement training. Accordingly, we believe the investigation on the passive case still is relevant and practical.

The TRR of *passive* movement has been reported by three research works (Estevez et al., 2014; Jaeger et al., 2015; Loubinoux et al., 2001). All of these commonly used fMRI, but the results are divided. To elaborate Loubinoux et al. (2001) examined the TRR of passive wrist movement for six subjects between two sessions separated by one month, and reported large across‐sessions variability in brain activation (Loubinoux et al., 2001), concluding essentially *no TRR*. Jaeger et al. (2015) investigated the TRR of passive stepping movements for 16 subjects between two sessions, 6 weeks apart, and showed highly variable TRR—from poor to excellent—across the subjects, leading to an *inconclusive* result. Estevez et al. (2014) examined the TRR of passive elbow movement for 19 subjects between two sessions at intervals of 3–4 weeks and reported TRR of high level at the primary sensory‐motor cortex, confirming the strong TRR.

Those divided results—one no‐TRR, one inconclusive, and one strong TRR—are already prompting to conduct a separate investigation, even more so after close examination of each of the aforementioned studies. Loubinoux et al. (2001) used the paired *t* test to evaluate the TRR. Using it alone for the TRR appears problematic, because it compares the *means* of a data set in one session with another in a different session, neglecting the individual across‐session difference, which really has to be assessed by the intraclass correlation coefficient (ICC) values (Bruton, Conway, & Holgate, 2000). The research result (Zaki, Bulgiba, Nordin, & Ismail, 2013) is alarming that the TRR—based on some hypothetical data—by the paired *t* test was *contradicted* by that of the ICC. Furthermore, the paired *t* test is plagued with the traditional problem of statistical power with a low sample size (Bedard, Martin, Krueger, & Brazil, 2000). Besides, it is noticeable that the three studies used *end‐effector* type mechanism or robots, which are prone to various kinds of motion artifacts. For instance, Jaeger et al. (2015) reported motion artifacts that contaminated the data of eight subjects—out of 16. The robot in Estevez et al. (2014), lacking devices to fix wrist, upper arm, and shoulder, is very likely to induce their voluntary movements, not the targeted elbow motion alone. In addition, the manual traction mechanism in Loubinoux et al. (2001), while trying to achieve accuracy in the period and frequency, is liable to suffer from inaccuracy and inconsistency.

The examination of the research above has made plain our research direction: the use of an exoskeleton robot along with the ICC measure. In addition, we are going to use an fNIRS instead of fMRI, considering the direction of our ongoing research: to investigate the additional effect of video games on brain activation, which would be extremely difficult to include under the MRI environment. Furthermore, while the fNIRS has lower spatial resolution than the fMRI (Boas, Dale, & Franceschini, 2004), it is less sensitive to motion artifacts and metallic materials, compared to other functional neuroimaging techniques (Bae, Jang, Seo, & Chang, 2017; Li, Inoue, Liu, & Sun, 2013; Mihara, Yagura, Hatakenaka, Hattori, & Miyai, 2010).

To this end, we have incorporated an in‐house developed exoskeletal robot having a capability to consistently provide accurate movements under high resistance torque, an important capability for clinical rehabilitation (Butefisch, Hummelsheim, Denzler, & Mauritz, 1995; Kwakkel, Kollen, & Lindeman, 2004; Murphy & Corbett, 2009; Oujamaa, Relave, Froger, Mottet, & Pelissier, 2009). In order to evaluate the TRR, we have employed an ICC, one of the most widely used index for reliability in neuroimaging study (Bhambhani et al., 2006; Estevez et al., 2014; Li, Zeng, Lin, Cazzell, & Liu, 2015; Plichta et al., 2007; Wiggins, Anderson, Kitterick, & Hartley, 2016). Hence, while this robot offered passive movements to healthy subjects, the brain activation was to be measured by using an fNIRS system, from which the TRR was examined. In this process, the robot was made to provide three different velocities (slow, moderate, and fast) in order to investigate their effects on the brain activation and the TRR, out of our experience from a previous similar study that velocity difference matters to brain activation patterns (Bae et al., 2017; Jang et al., 2015).

## 2 METHODS

### 2.1 Selection of the number of subjects and number of repeated sessions

For the evaluation of the TRR, previous studies set the number of sessions first—usually, *two*—and then tried to include as many subjects as possible; in this study, however, we set the number of sessions first and then endeavored to involve as few subjects as possible. As a result, we involved 10 repeated sessions on five subjects, which has the same statistical power as two repeated sessions on 50 subjects (Donner & Eliasziw, 1987). Details of the statistical testing method are described below.

We used the power contour of the number of sessions (*n*) and subjects (*k*) proposed by Donner and Eliasziw (1987) (Donner & Eliasziw, 1987). The power contour displays the required numbers of *n* and *k* based on a certain statistical power where the parameter of interest is the ICC. The ICC, an indicator of TRR, has a value between 0 and 1, and the closer to 1 means the higher reliability (Fleiss, 2011). A more detailed description is provided in Section 2.6.2.

Donner and Eliasziw (1987) calculated *n* and *k* for testing null hypothesis (*H*_0_: *ρ* = *ρ*_0_) and alternative hypothesis (*H*_1_: *ρ* > *ρ*_0_) at a chosen level of significance *α* and with statistical power 1−*β*. In other words, by setting the values of four parameters (*ρ*, *ρ*_0_, *α*, and 1−*β*), we can get the power contour of *n* and *k*. More specifically, *ρ* is the value of ICC that experimenters expect and *ρ*_0_, the minimum value of *ρ*, is heuristically determined by experimenters according to their judgment (Donner & Eliasziw, 1987). The value of *ρ* was determined according to the evaluation criteria reported in previous studies. Fleiss (2011) presented the evaluation criteria for ICC values as follows: poor (below 0.4), fair to good (between 0.4 and 0.75), and excellent (above 0.75), and also, Li et al. (2015) suggested similar criteria: poor (below 0.4), fair (between 0.4 and 0.6), good (between 0.6 and 0.75), and excellent (above 0.75) (Fleiss, 2011; Li et al., 2015). The value of *ρ* is set to 0.4 as the expected ICC value, which is the boundary between poor and fair levels, so we can check for the value of TRR. Since there had been no report as to *ρ*_0_ we could refer to, we had to resort to a conservative value, the minimum of *ρ*, which is 0. Regarding *α* and 1−*β*, we employed a generally accepted significance level (*α* = 0.05) and the statistical power (1 – *β* = 0.80) (Cohen, 2013). Based on these three values (*ρ* = 0.4, *ρ*_0_ = 0, *α* = 0.05, and *β* = 0.20), the power contours can be obtained.

**FIGURE 1:**
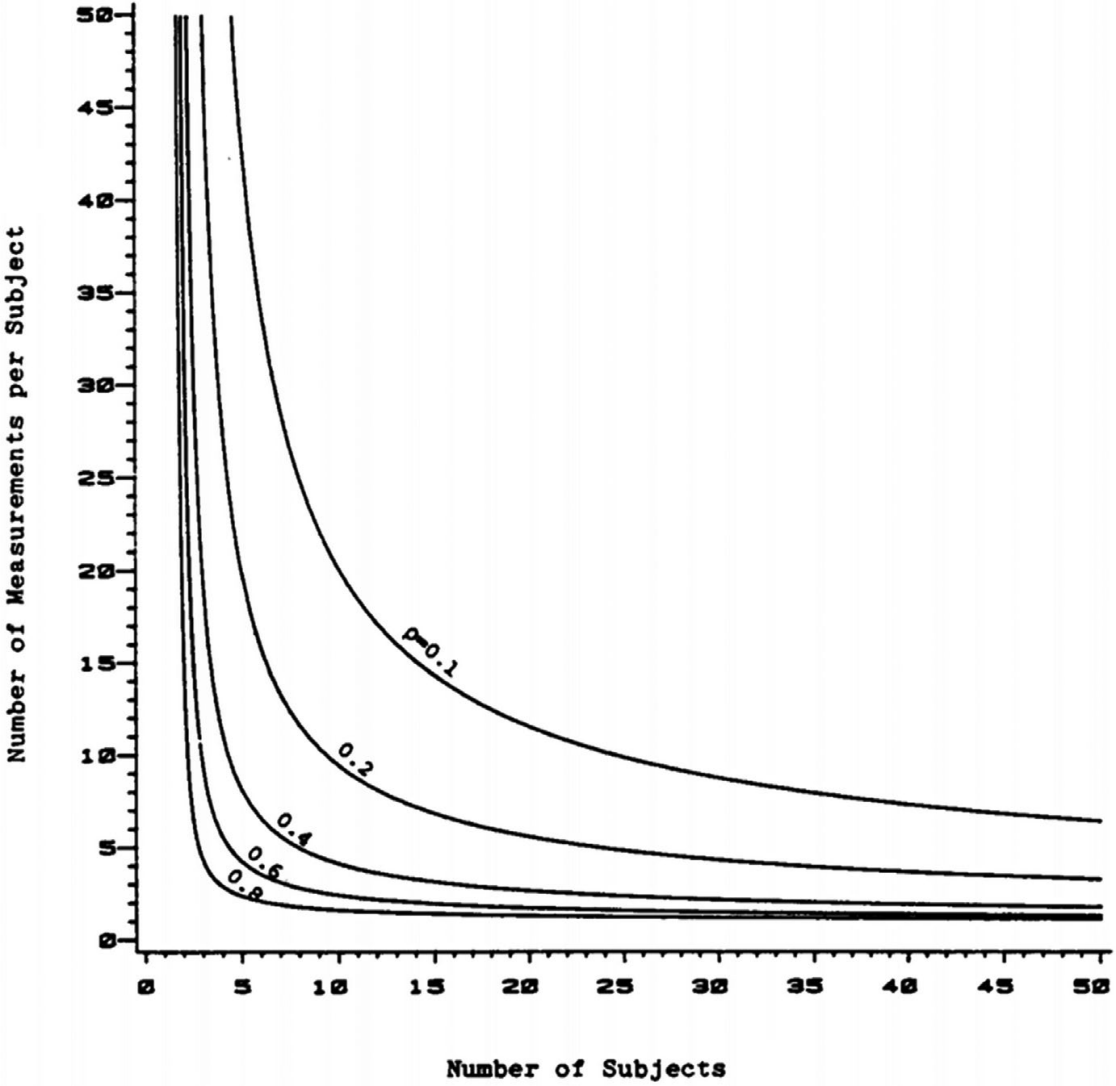
The power contours of the number of subjects (*k*) and the number of sessions (*n*) for *ρ* = 0, 0.2, 0.4, 0.6, 0.8 (Donner & Eliasziw, 1987). It shows the values of *k* and *n* for testing (*H*_0_: *ρ* = *ρ*_0_) versus (*H*_1_: *ρ *> *ρ*_0_) at a chosen level of significance *α* = 0.05 and with statistical power 1−*β* = 0.80

Figure 1 shows a group of power contours of *n* and *k* for several values of *ρ* (0.1, 0.2, 0.4, 0.6, and 0.8), with each contour being determined by the value of *ρ* and representing different sets of *n* and *k* that have the same statistical power (Donner & Eliasziw, 1987). We selected (*n* = 10, *k* = 4.31) from the power contour because we were interested in the effect of many sessions and various intervals on TRR. Finally, we decided to perform 10 repetitive sessions in five subjects.

### 2.2 Subjects

Since there was no previous study on the TRR of brain activation by robotic passive hand movement, we decided to test *normal* people, before a clinical test. Five healthy subjects participated in the experiment without any history of neurological, psychological, and physical illnesses (four males, average age: 21.8, range 21–23). All subjects were confirmed to be right‐handed by using Edinburg Handedness Inventory (Oldfield, 1971). Before the experiment, all subjects were fully informed about the purpose of the research and provided written, informed consent. This study was approved by the Institutional Review Board of Daegu Gyeongbuk Institute of Science and Technology.

### 2.3 Robotic passive hand movement

As was mentioned, the robotic passive hand movement was selected as an external stimulus to induce brain activation. The selection of the hand part as the target was out of the consideration that it is in the hands where the loss of motor function is prevalent in stroke patients (Fischer et al., 2007). The finger extension, in particular, is easily damaged, making daily activities (e.g., button‐down shirt, picking up cups) difficult (Cauraugh, Light, Kim, Thigpen, & Behrman, 2000; Radomski & Latham, 2008). For this reason, flexion and extension of four fingers were selected as the target movement. This movement was provided for all the subjects under the same experimental condition (range of movement, velocity, and the number of repetitions).

**FIGURE 2:**
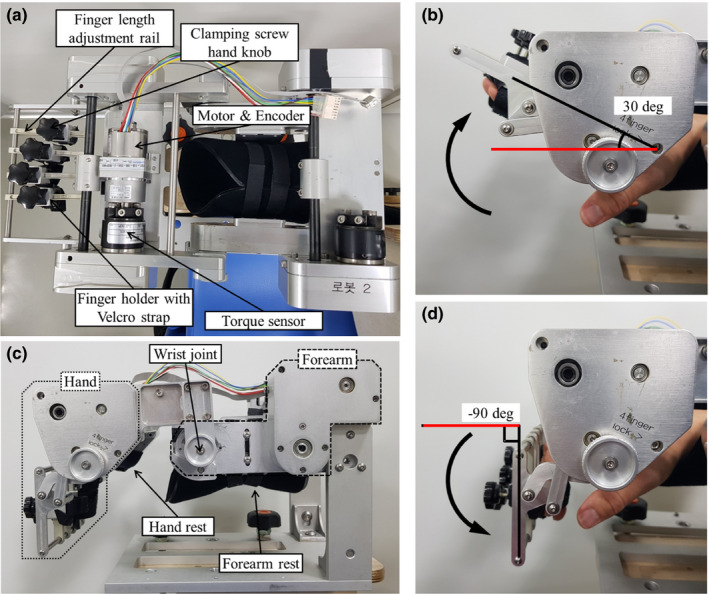
(a) Top view of hand robot; it mainly shows the actuation part and finger holding part. The clamping screw can be used to fix or position the finger holder on the length adjustable rail. (b) Left lateral view of finger extension (30 deg). (c) Left lateral view of hand robot; hand rest and forearm rest support hand and forearm, respectively. (d) Left lateral view of finger flexion (−90 deg)

That flexion/extension was realized by the kinematic mechanism, driven by actuators–sensors under the supervision of the control system, each of which is detailed in the following. The simultaneous flexion/extension of the four fingers was implemented in one degree of freedom motion produced by a four‐bar linkage mechanism. This mechanism has been synthesized so that it can accurately track a predefined trajectory generated by actual finger movement (Chang et al., 2014). Figure 2a,c show the top and left lateral side of the hand robot, respectively. The hand robot consists of two parts: the hand part and the forearm part (Figure 2c). The former provides 30 degree extension (Figure 2b) and −90 degree flexion (Figure 2d) with the finger attached to the finger holder and with the palm supported by the hand rest, whereas the forearm was supported by the forearm rest (Figure 2c). The actuators/sensors comprise a brushless DC motor with an encoder (EC‐i 40, Maxon motor, nominal torque 43.3 mNm), harmonic drive (CSF‐11‐50, Sam‐ik THK, gear ratio 50:1), and force–torque sensor (TRT 100, Transducer Techniques; capacity range 11.30 Nm). The control system consists of a hardware platform and a real‐time control system. The hardware platform consists of an industrial PC (Intel core i3‐3240) and a Sensoray s626 board. A real‐time control system consisting of Linux Ubuntu 16.04 LTS with Linux kernel 4.1.18 and Real‐Time Application Interface for Linux (RTAI) Ver 5.0.1 systems was installed on this hardware platform. In this environment, the time delay control (TDC) was implemented with a sampling time of 0.002 s (Youcef‐Toumi & Ito, 1988).

In addition, the TRR was examined at three different velocities (slow, 0.25 Hz; moderated, 0.5 Hz; and fast, 0.75 Hz), according to previous studies reporting that brain activation became different depending on the three velocities (Bae et al., 2017; Jang et al., 2015).

### 2.4 fNIRS measurement

In this study, brain activation was measured by an fNIRS system (LABNIRS; Shimadzu) with continuous near‐infrared light of three wavelengths (780, 805, and 830 nm) at a sampling rate of 27 Hz. After measuring the optical density change by using the fNIRS system, we obtained the relative changes in concentration of oxygenated hemoglobin (oxy‐Hb) and deoxygenated hemoglobin (deoxy‐Hb) according to the modified Beer–Lambert law, and then, the brain activation was evaluated (Cope & Delpy, 1988; Delpy et al., 1988). According to many previous findings that the oxy‐Hb is the most sensitive to task‐related hemodynamic changes, we have selected it as an index to assess the brain activation (Hoshi, Kobayashi, & Tamura, 2001; Strangman, Culver, Thompson, & Boas, 2002; Suzuki et al., 2004; Wolf et al., 2002).

For the measurement, after the ROI was set in the cerebral cortex, the optode holder cap was placed on the head of the subject, and then, the NIRS optodes were arranged to cover the ROI. The primary sensorimotor cortex (SM1; BA1, 2, 3, and 4) of the left hemisphere was selected as the ROI considering both Brodmann's area (BA) and the anatomical locations of the brain (Martin, 2012; Mayka, Corcos, Leurgans, & Vaillancourt, 2006).

**FIGURE 3:**
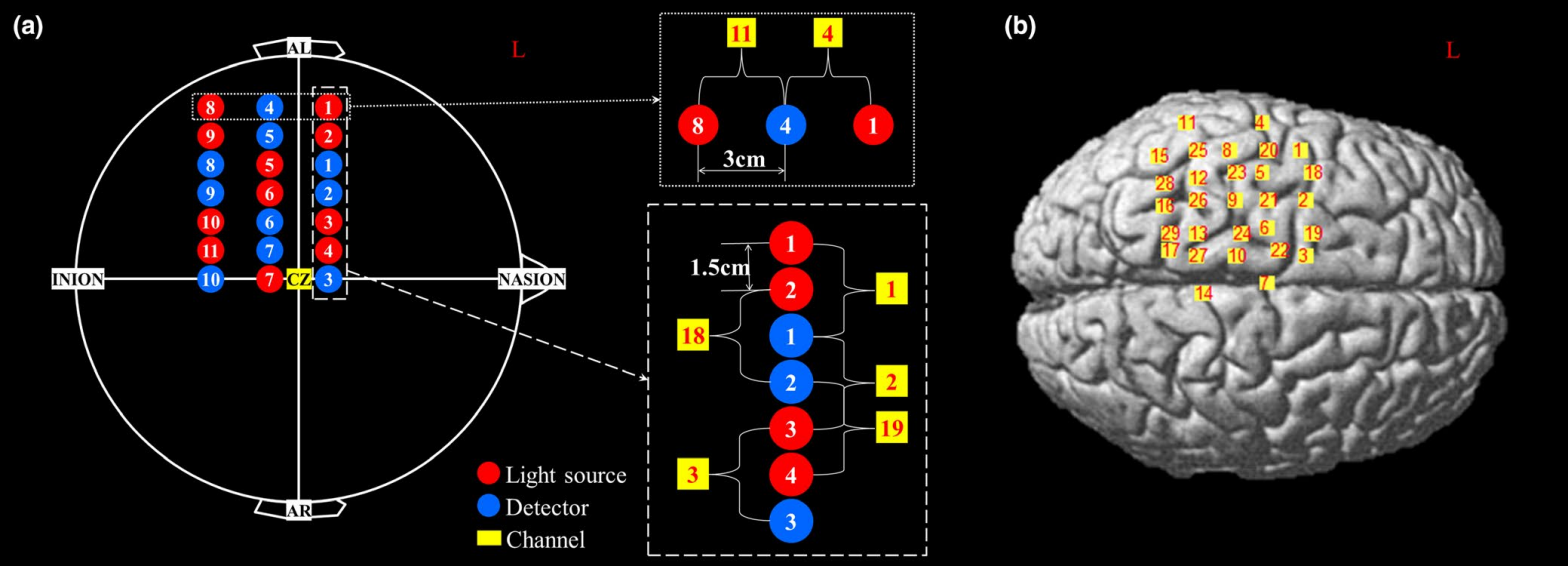
(a) The arrangement of NIRS optodes from the dorsal view of the brain. Twenty‐one NIRS optodes (11 light sources and 10 detectors) are arranged in a seven by three rectangular arrangement on the left side of the brain. Each of the seven rows has three optodes being 3 cm apart, and each column places seven optodes with 1.5 cm apart. Each channel is created in the center between the source and detector only if the source–detector (*SD*) distance is 3 cm. (b) The location of 29 measurement channels

We used the international 10–20 system and the anatomical landmark positions, such as cranial vertex (CZ), left/right preauricular points (AL/AR), Nasion, and Inion, in order to position the optode holder cap on the head for each subject (Jasper, 1958). Specifically, first, by using a measuring tape and marking pencil, we measured the length from the Nasion to Inion over the center line (see Figure 3a) of the scalp and marked 50% of it to find CZ. Then, we measured the length from AL to AR and marked 50% of it and reconfirm the position of CZ on the same point. As shown in Figure 3a, we set Cz as a reference point and two perpendicular lines (one connecting Nasion/Inion and the other connecting the AL/AR) as reference lines to locate the optode holder cap.

In order to cover the whole ROI, 21 NIRS optodes (11 light sources and 10 detectors) were arranged in a double‐density (DD) arrangement provided by Shimadzu fNIRS system, measuring brain activation in a total 29 channels (Figure 3a,b). In DD arrangement, optodes are more densely arranged than the conventional arrangement, for more measurement channels and spatial resolution (Ishikawa et al., 2011; Yamamoto et al., 2002). Note that the DD and conventional arrangement have the same source–detector (*SD*) distance of 3 cm for measuring brain activation, but the distance between optodes is slightly different. As shown in Figure 3a, 21 NIRS optodes are arranged in seven by three matrix arrangement. Each of the seven rows has three optodes placed 3 cm apart, and each of the three columns places seven optodes with 1.5 cm apart. As a result, a total of 29 channels are created in the center only if the source–detector (*SD*) distance is 3 cm (Figure 3b). After placement of the optodes, the anatomical landmark positions of the international 10–20 system and all the 3D coordinates of the optodes were measured by using the 3D digitizer of the Fastrak System (TX‐2; Polhemus) (Okamoto et al., 2004).

### 2.5 Test protocol

**FIGURE 4:**
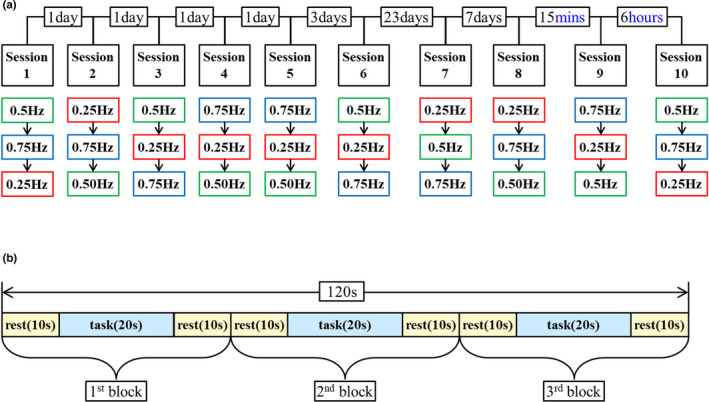
(a) Schematic representation of experimental protocol; it describes interval, sessions, and three velocities from the top line. The order of three velocities is random in each session. (b) Schematic representation of the block design paradigm. During the triple repetition of a block consisting of a 10‐s rest, a 20‐s task, and a 10‐s rest, the brain activation was measured for each velocity

In this section, we describe the experimental conditions, procedures, and instructions for the subjects employed for the evaluation of the TRR of brain activation. As is illustrated in Figure 4, we have equally applied to all the subjects the three experimental conditions: *session intervals*, the *three velocities* in each session, and the *block design* for each velocity.

As to the session intervals, we set the following intervals: 1 day, 3 days, 23 days, 7 days, 15 min, and 6 hr, as is shown in Figure 4a. Cumulatively, there were four daily sessions after the first session, 1 week after it, 1 month, 37 days, and so on. This somewhat irregular interval setting needs some explanation. The original intervals had been set as follows: 1 day (four times), 3 days, 23 days, 1 month (twice), and 3 months; cumulatively, 1 day, 1 week, 1 month, 2 months, 3 months, and 6 months, all after the first session. We had chosen these long intervals in order to observe long‐term effects that rehabilitation generally tends to involve. Since no TRR had been observed until 1 month, however, we made a decision to modify the remaining three intervals to 1 week, 15 min, and 6 hr. Compared with the intervals in the previous studies, which were set rather arbitrarily ranging from 15 min (Strangman et al., 2006) to 6 months (Sato et al., 2006) (Sato et al., 2006; Strangman et al., 2006), our interval setting fills in‐between.

In each session, the robotic movements with three different velocities were exerted in random orders to all the patients, while the measurement is performed once for each velocity. For we were wondering if the velocity difference made any difference in the TRR, just as it had done in the brain activation in the previous studies (Bae et al., 2017; Jang et al., 2015). The random orders were generated by using the random permutation function “randperm” of MATLAB R2012b (The MathWorks) so that the order of measurement did not affect the results (Figure 4a).

These three velocities were exerted, and their corresponding activations were measured by using the block design paradigm (Maki et al., 1995). Specifically, for each velocity, brain activation was measured during the triple repetition of a block consisting of a 10‐s rest, a 20‐s task, and a 10‐s rest (Figure 4b). Thus, the three velocities took nine such blocks for each subject in a session.

The details of the test procedure are chronologically described together with the instructions for the subjects. As a subject entered an electromagnetically shielded room with minimal noise and light, he or she was ordered to sit down in a rehabilitation chair that can hold the body part as needed. After the trunk was fixed to the chair with a strap to prevent the trunk movement, the subject was instructed to wear the robot on his or her right hand, together with verbal instruction, “please sit as comfortable as possible and relax.” After placing the optode holder cap on the subject's head and inserting the optodes into the cap, the preparation became completed for the measurement. A few instructions were given before the measurement started, such as “look at the front wall,” and “do not voluntarily move the body parts including the right hand during the measurement.”

Motion artifacts can easily be generated by the movement of the subject (especially head), causing a decoupling of optodes from the scalp, which affects the measured signal (Brigadoi et al., 2014; Cooper et al., 2012). Usually, motion artifacts in NIRS data are relatively easy to identify by observation of the subject during NIRS recording (Cooper et al., 2012). Therefore, in the measurement phase, whether the subject made spontaneous body movement was closely monitored by two operators. Further, the irregular nature of the motion artifacts was contrasted by the regular movement by the robot, becoming far easier to discern. After the experiments, we reconfirmed from all the subjects that there had been no voluntary movement.

### 2.6 Data analysis

This Section describes the processing of the measured fNIRS data and their statistical analysis for the TRR of brain activation induced by robotic passive hand movement.

#### 2.6.1 Analysis for fNIRS

For data processing of the fNIRS signal, we used NIRS‐SPM (Near Infrared Spectroscopy‐Statistical Parametric Mapping; http://bisp.kaist.ac.kr/NIRS‐SPM), a MATLAB‐based software package (Ye, Tak, Jang, Jung, & Jang, 2009). The signal processing was performed in roughly four stages. In the first stage, spatial registration of 3D coordinates of channels measured by the 3D digitizer was performed to the standard brain space of the Montreal Neurological Institute (Singh, Okamoto, Dan, Jurcak, & Dan, 2005). It maps the measured channels to the standard brain.

In the second stage, we have preprocessed the fNIRS signal to remove the unwanted noise caused by task‐related skin blood flow, motion artifact, and physiological oscillations.

First, task‐related skin blood flow was removed by applying the *independent component analysis* (ICA) along with the *coefficient of spatial uniformity* (CSU). To elaborate, by using the built‐in software of LABNIRS, we applied the Molgedev and Schuster‐ICA (Molgedey & Schuster, 1994) on raw data, with time delays between 0 and 0.74 s with steps of 0.037 s, to fNIRS signals of 29 channels. As a result, we obtained the independent oxy‐Hb components and the mixing matrix, both of which were used to calculate the CSU value for each independent oxy‐Hb component (Kohno et al., 2007). This CSU value determines the task‐related skin blood flow. Specifically, Kohno et al. (2007) regarded the independent components with high CSU value as the task‐related skin blood flow. As such high CSU values, we set the one equal or higher than 1.5 and removed independent component with high CSU value from the fNIRS signal (Seiyama, Higaki, Takeuchi, Uehara, & Takayama, 2016).

Second, noises due to motion artifacts and physiological oscillations were eliminated by appropriate filters embedded in the NIRS‐SPM (Ye et al., 2009): Gaussian smoothing with a full width at half maximum of 2 s (Worsley & Friston, 1995) as a low‐pass filter and wavelet‐minimum description length detrending algorithm (Jang et al., 2009) as a high‐pass filter.

In the third stage, the general linear model (GLM), one of the linear regression model, was used for statistical analysis to infer the brain area that was significantly activated during the robotic passive hand movement (Friston et al., 1994). The GLM is defined by the following Equation 1:

(1) Y=Xβ+ε

where ***Y*** denotes the vector of measured oxy‐Hb data in time series. ***X*** stands for a design matrix that is a convolution of the canonical hemodynamic response function and block design function, which means the expected oxy‐Hb response under our block design condition. ***ε*** represents the vector of measurement error. ***β*** denotes the parameter vector, which corresponds to the regression coefficient of GLM and means the amplitude of the oxy‐Hb reaction. Through the least square estimation, ***β*** is obtained so that ***ε*** may be minimized for each channel.

In the fourth stage, we are to infer the significantly activated area by obtaining individual *t*‐statistics maps using the ***β*** for each subject. At this time, individual *t*‐statistics maps are obtained through a *t* test that tests the null hypothesis that ***β*** is 0 (meaning no significant brain activation) for each subject (Ye, Tak, Jang, Jung, & Jang, 2009). At this time, ***t* statistics of *β*** was used as an index for verifying the significance of brain activation at the level of *p* < .05. The Lipschitz–Killing curvature‐based Euler characteristic (EC) approach was used to control the familywise error rate resulting from multiple statistical hypothesis tests (Li, Tak, & Ye, 2012).

#### 2.6.2 Linear mixed effect model and ICC

In this study, we investigated the effect of the variables of interest (session intervals, robot velocities, subject, and session) on brain activation and TRR through the linear mixed effect model and ICC.

In order to perform statistical analysis on repeated measurement data, we have selected a linear mixed effect model (LMM). The LMM was particularly useful to our study for the following reasons: Firstly, we can set up the relationship of the ***t* statistics of *β*** to various variables of interest such as subjects, sessions, intervals, and robot velocities. Secondly, we can easily appropriate these variables to either the fixed effect or random effect. Finally, as a direct outcome of the second reason, assigning the subject to the random effect enables us to analyze the *within‐subject correlation*, correlation data from different sessions of the same subject (Gelman & Hill, 2006; Laird & Ware, 1982; Littell, Pendergast, & Natarajan, 2000).

The LMM used in this study can be written as

(2) $$\begin{array}{c} y_{\mathrm{ijkl}}\;=\;\mu \;+\;\alpha_{k}\;+\;\gamma x_{j}\;+\;b_{i}\;+\;b_{\mathrm{ij}}\;+\;\varepsilon_{\mathrm{ijkl}}\\ i\;=\;1,\ldots ,5\\ j\;=\;1\;\text{day},1\;\text{day},1\;\text{day},1\;\text{day},3\;\text{days},23\;\text{days},7\;\text{days},0.01047\;\text{day}\;(=15\;\min),0.25\;\text{day}\;(=6\;\text{hr})\\ k\;=\;0.25\;\text{Hz},0.5\;\text{Hz},0.75\;\text{Hz}\\ l\;=\;1,\ldots ,29 \end{array}$$

$$b_{i}\;∼\;N\left(0,\sigma_{\text{subject}}^{2}\right),\;b_{\mathrm{ij}}\;∼\;N\left(0,\sigma_{\text{session}\;\text{nested}\;\text{by}\;\text{subject}}^{2}\right),\;\varepsilon_{\mathrm{ijkl}}\;∼\;N\left(0,\sigma_{\text{error}}^{2}\right)$$

where *y_ijkl_*, the dependent variable, is the ***t* statistics of *β*** (see Section 2.6.1) at the session *j* for channel *l* of subject *i* under the condition of velocity *k*. *μ* is a grand mean of all *y_ijkl_*. *α_k_* is a vector of fixed effect for velocity and *x_j_* is a vector of fixed effect for the interval between *j* th session and *j*+1 th session, and *γ* is the vector of regression coefficients. The two intervals, 15 min and 6 hr, were converted to 0.01047 and 0.25 day, respectively, with 1 day being used as its standard unit. *b_i_* is a vector of random effect for subjects, *b_ij_* is a vector of nested random effect for sessions within a subject, and *ε_ijkl_* is the residual error. The random effects *b_i_* are independently distributed from $N\left(0,\sigma_{\text{subject}}^{2}\right)$ and *b_ij_* are independently distributed from $N\left(0,\sigma_{\text{session}}^{2}\right)$. Also, *ε_ijkl_* are independently distributed from $N\left(0,\sigma_{\text{error}}^{2}\right)$. The statistical analysis of LMM was performed by lmer function in the lme4 package (Bates, Maechler, Bolker, and Walker 2014a) and other packages (e.g., dplyr, base, rJAva, xlsxjars, and stats) supplied in the R system for statistical computing (Ver. 3.4.4; R Development Core Team, 2018).

To quantify the TRR of brain activation, we used the ICC. Conceptually, the ICC represents the proportion of the total variance in the measurements which is due to between‐subject differences (Streiner et al., 2015). In this study, the ICC can be defined as follows:

(3) $$\text{ICC}\;=\;\sigma_{\text{subject}}^{2}/\sigma_{\text{subject}}^{2}+\sigma_{\text{session:subject}}^{2}+\sigma_{\text{error}}^{2}$$

In this equation, $\sigma_{\text{subject}}^{2}$ means between‐subject variance, $\sigma_{\text{session:subject}}^{2}$ means between‐session variance nested by subject, and $\sigma_{\text{error}}^{2}$ means the variance of residual error. ICC close to 1 represents high TRR and occurs when the between‐subjects variance is much larger than the variance of others (Johnstone et al., 2005).

## 3 RESULTS

This Section reports three main results. First is the activation map of oxy‐Hb, which shows how the brain activation induced by robotic passive hand movement changes according to the session and velocity of robot. The second is the result of LMM statistical analysis, which confirms the effect of the variables (interval, velocity of robot, subject, and session) of interest on brain activation and TRR. The third is the ICC value for evaluating TRR.

**FIGURE 5:**
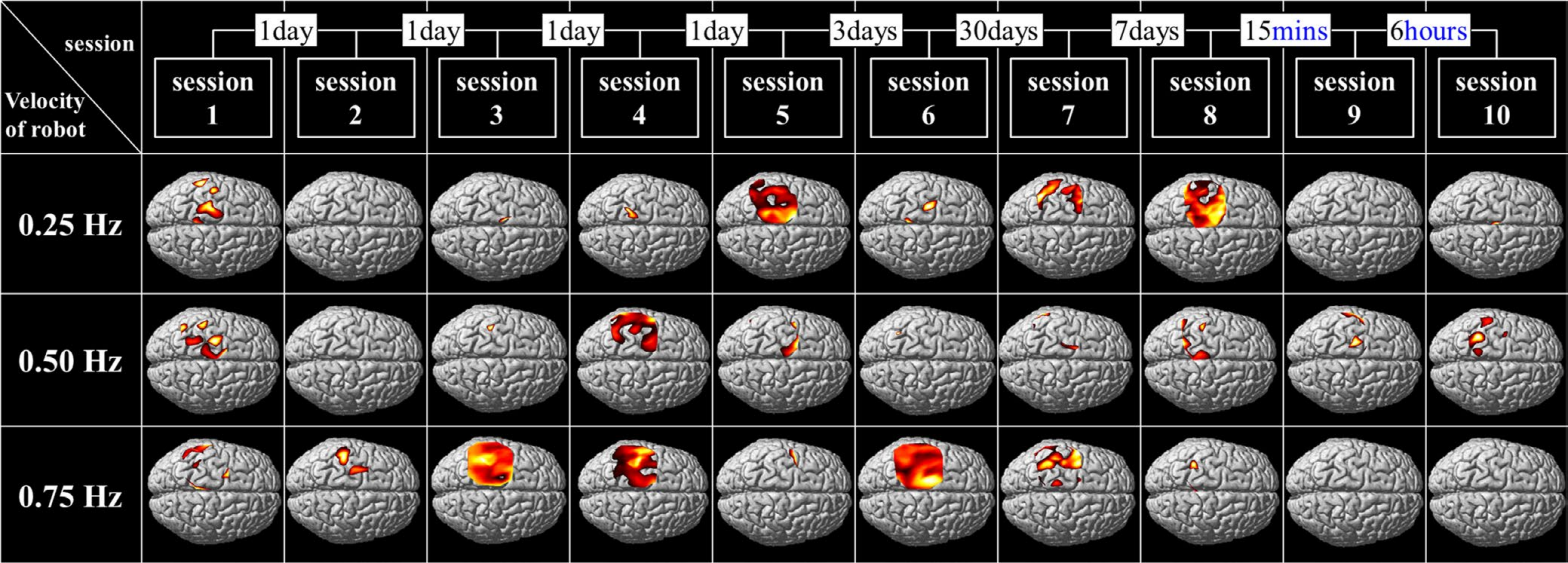
Activation maps of one subject according to velocity and session. The intervals between 2 consecutive sessions are shown in the top row. The highlighted area showed significant activation at the level of *p* < .05

As a first result, Figure 5 shows the activation map of one subject, displaying activation areas, if there is any, according to a session and velocity. The highlighted area showed significant activation at the level of *p* < .05. An activation map with no highlighted area means there is no significantly activated area. Clearly a trend was observed that a velocity that induced significant activation of SM1 in one session could not do so in other sessions. More generally, session‐to‐session variation is prominent in the existence of activation, in its intensity, and in its area.

As a second result, Table 1 summarizes the LMM analysis, showing the effect of the fixed effect (velocity of the robot (*α_k_*) and interval (*γ*)) on ***t* statistics of *β*** (see Section 2.6.1).

**TABLE 1:** Summary of LMM results examining ***t* statistics of** ***β*** in relation to the fixed effects (velocity and interval)

Fixed effects	Estimate	Standard error	*T* value
Velocity			
*α*_0.25 Hz_ (intercept)	−0.075	0.117	−0.641
*α*_0.5 Hz_	−0.304	0.107	−2.833
*α*_0.75 Hz_	0.072	0.107	0.669
Interval			
*γ*	0.019	0.010	1.864

The **estimate** of *α_k_* and *γ* of the LMM, according to Equation 2, corresponds to the **estimate** of velocity and interval in TABLE 1. The ***T* value** in this table is used to determine the significance level and the relationship between the fixed effect and the ***t* statistics of *β***. If the absolute ***T* value** is greater than 1.96, the fixed effect is considered statistically significant at the significance level of 5% (Gelman & Hill, 2006). With respect to the effect of velocity, the ***t* statistics of *β*** is significantly smaller at 0.5 Hz than 0.25 Hz and 0.75 Hz. However, the effect of interval on the ***t* statistics of *β*** was not significant.

As the third result, an ICC value of 0.002 was obtained. To calculate the ICC value, the variance of random effects (subject (*b_i_*), session nested by subject (*b_ij_*), and residual error (*ε_ijkl_*)) were used (Table 2). The ICC value of 0.002 means poor TRR according to the guide interpretation of ICC described above.

**TABLE 2:** Summary of LMM of random effects. ICC was calculated by using the variance of each effect

Random effects	Variance	Standard deviation
Session nested by subject		
*b_ij_*	$\sigma_{\text{session:subject}}^{2}$: 0.149	0.387
Subject		
*b_i_*	$\sigma_{\text{subject}}^{2}$: 0.013	0.115
Residual error		
*ε_ijkl_*	$\sigma_{\text{error}}^{2}$: 7.506	2.740

## 4 DISCUSSION AND CONCLUSION

We began this study with the question: “Does the brain activation induced by the robotic passive movement have TRR?” The answer is *negative*. *Poor* TRR was confirmed, based on the activation map and ICC value. In other words, repeated activation of an area is not achievable with the *passive* robotic movement, and brain plasticity cannot be expected. This result is in contrast to those of active hand movements. Note that the passive movement training by *human* therapists—no clinical study has reported yet—is unlikely to produce any better result. For what the subjects passively receive is essentially the same movement, whether from a therapist or from a robot; though the former tends to lack the accuracy and consistency, the latter possesses in generating the movement.

Three studies examined the TRR of passive movement and reported divided results; Loubinoux et al. (2001) reported poor TRR (Loubinoux et al., 2001), while another (Estevez et al., 2014) observed TRR of high level at the SM1 area, the ROI; and still, another (Jaeger et al., 2015) showed inconclusive results, with some subjects showing good TRR and others showing poor TRR. Obviously, these divided results are directly attributable to substantially variable brain activations, which in turn could be explained as the outcome of flawed tests coming from the motion artifacts or the misleading interpretation lacking the ICC, mentioned in Introduction. The fact that those tests using fMRI with the highest spatial resolution could have generated such different results is noteworthy, underlining the importance of controlling experimental attributes.

Compared to the previous studies, the ICC value we have obtained is 0.002, an order of about 1/100 times smaller, which could be viewed as a more *definitive* result (Estevez et al., 2014; Jaeger et al., 2015). Nevertheless, what it means to have that ICC value generated by a *low‐resolution‐tester* like fNIRS is open to questions, prompting further investigation. Our study, too, has observed a high level of across‐session variability in brain activation. The variability may be ascribed to the weakness of the stimulus, the characteristics shared by any passive movement. It could also have come from the *familiarity* with the fNIRS environment, diminishing the attention and affecting brain activation (Loubinoux et al., 2001). The familiarity factor could have been severe in our experiments, since a 10 times repetition of the same passive movements was conducted by the participants.

What causes then the difference between the passive movement and the active one in the TRR? The main difference, we believe, lies in the presence of *active user engagement* in the active movements (Blank, French, Pehlivan, & O'Malley, 2014; Weiller et al., 1996), which explains the recent emphasis on its importance and its enhancement (Blank et al., 2014; Krebs, Volpe, & Hogan, 2009). User engagement is defined as effortful striving toward task goals and is known to be affected by mental effort, motivation, and affective status (Fairclough, Gilleade, Ewing, & Roberts, 2013). Previous studies proved the effect of user engagement to active movement training (Ferraro et al., 2003; Krebs et al., 2009). The active engagement theory has been further supported by a study reports that it induces in a rat model the neural plasticity in motor learning (Warraich & Kleim, 2010). However, the user engagement in passive movement thought to be low because it is achieved involuntarily by the therapist or robot. In addition, disengagement can also occur if the subject becomes easily bored and indifferent to simple repetitive passive movement (O'Brien & Toms, 2008). Considering these encouraging results and the still existing need for passive movement training for some patients, one may consider to take a course that attempts a passive training involving the active engagement.

What is necessary to induce TRR in passive movement? Two such approaches have already been underway. The one is to provide visual, auditory, or tactile cues that encourage active engagement during passive hand movement (Blank et al., 2014). The other is to apply motor imagery practice which refers to imagining without physical movement to learn or improve motor ability (Denis, 1985). The previous study reported that physical therapy combined with motor imagery practice for stroke patients improves motor function and ability (Page, 2000). Song, Oh, Jeong, Kim, and Kim (2018) developed a brain–computer interface system that detects movement intention through motor imagery and provides robotic passive movement (Song et al., 2018). Hopefully, we like to be able to see more progress in robotic passive movement protocols enhanced with active engagement that target the TRR, and ultimately brain plasticity.

Unlike the previous studies, we decided the number of sessions (*n* = 10) first before the number of subjects (*k* = 5), by using the power contour based on the statistical power (Donner & Eliasziw, 1987). Table 3 shows that all of the previous studies involved two sessions, whereas the number of subjects varied from two to 25 (Bhambhani et al., 2006; Durduran et al., 2004; Plichta et al., 2007; Sato et al., 2006; Strangman et al., 2006). In comparison, our combination, *n* = 10 and *k* = 5, has a statistical power equivalent to *n* = 2 and *k* = 50 in the previous studies. Even if the method by Donner and Eliasziw (1987) is a statistical approach that is mathematically true and universally applicable, it is still necessary to conduct a crosscheck in the future to confirm if the same result is obtained with this combination (*n* = 2 and *k* = 50). If it is confirmed the implication may be very significant in many experimental studies including the one on the TRR of brain activation, offering useful and convenient alternatives between the number of sessions and the number of subjects.

**TABLE 3:** The number of subjects and intervals between two sessions in previous studies investigating the TRR of brain activation induced by active hand movement using fNIRS

Author (year)	Number of subjects	Number of sessions	Interval
Plichta et al. (2007)	12 (normal)	2	3 weeks
Durduran et al. (2004)	2 (normal)	2	2 weeks
Bhambhani et al. (2006)	13 (normal)	2	2 days
	25 (patient)		
Sato et al. (2006)	7 (normal)	2	6 months
Strangman et al. (2006)	19 (normal)	2	15 min

Although the main topic of this study was to examine the TRR, some additional understanding has been obtained as to how the brain *activation* is related to robot velocity and session interval, respectively. More specifically, the significance of brain activation being represented by ***t* statistics of *β*** as displayed in Table 1, its comparison exhibits the respective effect of velocity and interval on the activation. As to the velocity, 0.5 Hz has a significantly lower activation than that of other velocities, enabling us to deduce that there exists no linear relationship between velocity and activation. As to the interval, it has no significant relationship with brain activation. From these two points, one may draw a conclusion that both the velocity difference and interval are not critical for brain activation in the case of passive movement.

## CONFLICT OF INTEREST

All authors declare that there is no conflict of interests regarding the publication of this article.

## AUTHOR CONTRIBUTION

PC and SB conceived and designed the study. SB performed data collection and analysis. YL contributed to design the statistical model and statistical analysis. All authors contributed to data interpretation. PC and SB wrote the paper and critically revised the manuscript. All authors approved the final version of the manuscript.

### Peer Review

The peer review history for this article is available at https://publons.com/publon/10.1002/brb3.1788.

## Data Availability

The data for supporting the findings of this study are available in Mendeley Data with identifier “http://dx.doi.org/10.17632/sf3vh4grdf.3” (Bae, Lee, & Chang, 2020).
